# Spatiotemporal clustering of malaria in southern-central Ethiopia: A community-based cohort study

**DOI:** 10.1371/journal.pone.0222986

**Published:** 2019-09-30

**Authors:** Tarekegn Solomon, Eskindir Loha, Wakgari Deressa, Taye Gari, Bernt Lindtjørn

**Affiliations:** 1 School of Public Health, College of Medicine and Health Sciences, Hawassa University, Hawassa, Ethiopia; 2 Centre for International Health, University of Bergen, Bergen, Norway; 3 Department of Infectious Disease Epidemiology, London School of Hygiene and Tropical Medicine, London, England, United Kingdom; 4 Department of Preventive Medicine, School of Public Health, College of Health Sciences, Addis Ababa University, Addis Ababa, Ethiopia; Ministry of Health and Sports, MYANMAR

## Abstract

**Introduction:**

Understanding the spatiotemporal clustering of malaria transmission would help target interventions in settings of low malaria transmission. The aim of this study was to assess whether malaria infections were clustered in areas with long-lasting insecticidal nets (LLINs) alone, indoor residual spraying (IRS) alone, or a combination of LLINs and IRS interventions, and to determine the risk factors for the observed malaria clustering in southern-central Ethiopia.

**Methods:**

A cohort of 34,548 individuals residing in 6,071 households was followed for 121 weeks, from October 2014 to January 2017. Both active and passive case detection mechanisms were used to identify clinical malaria episodes, and there were no geographic heterogeneity in data collection methods. Using SaTScan software v 9.4.4, a discrete Poisson model was used to identify high rates of spatial, temporal, and spatiotemporal malaria clustering. A multilevel logistic regression model was fitted to identify predictors of spatial malaria clustering.

**Results:**

The overall incidence of malaria was 16.5 per 1,000 person-year observations. Spatial, temporal, and spatiotemporal clustering of malaria was detected in all types of malaria infection (*P*. *falciparum*, *P*. *vivax*, or mixed). Spatial clustering was identified in all study arms: for LLIN + IRS arm, a most likely cluster size of 169 cases in 305 households [relative risk (RR) = 4.54, P<0.001]; for LLIN alone arm a cluster size of 88 cases in 103 households (RR = 5.58, P<0.001); for IRS alone arm a cluster size of 58 cases in 50 households (RR = 7.15, P<0.001), and for control arm a cluster size of 147 cases in 377 households (RR = 2.78, P<0.001). Living 1 km closer to potential vector breeding sites increased the odds of being in spatial clusters by 41.32 fold (adjusted OR = 41.32, 95% CI = 3.79–138.89).

**Conclusions:**

The risk of malaria infection varied significantly between *kebeles*, within *kebeles*, and even among households in areas targeted for different types of malaria control interventions in low malaria transmission setting. The results of this study can be used in planning and implementation of malaria control strategies at micro-geographic scale.

**Trial registration:**

PACT R2014 11000 882128 (8 September 2014).

## Introduction

Malaria is a major global public health problem. In 2017, there were about 219 million malaria cases and 435,000 related deaths worldwide [1]. Among these, an estimated 92% of cases of malaria and 93% of deaths occurred in Sub-Saharan Africa [1]. In Ethiopia, 60% of the population is at risk, and 68% of the land is favorable for malaria transmission [2]. *Anopheles arabiensis* is the main malaria vector, and *Plasmodium falciparum* (60%) and *Plasmodium vivax* (40%) are the main malaria parasites in Ethiopia [2, 3]. Malaria transmission is seasonal and unstable in many parts of the country [2, 4, 5], occurring mostly between September and December, following the July and August rainfalls. Another smaller peak occurs in May and June, following short rains [6].

Over the last 15 years, considerable efforts (e.g., increased vector control, improved diagnosis and treatment) have led to a decline in malaria morbidity and mortality. The overall reduction in the global incidence of malaria is estimated at 37%, and the reduction in malaria-specific mortality is estimated at 60% [7]. Similar reductions have been observed in Ethiopia [3, 8]. However, despite these gains, control efforts remain inadequate, and malaria continues to be a major health problem [9].

Studies suggest that additional steps can be taken to further reduce malaria infection [10, 11], such as a more targeted intervention using available, though limited, resources in low to moderate malaria transmission areas [10, 12]. Studies have shown that 20% of a source population for infectious diseases could contribute to 80% of cases in the wider population, and such transmission often occurs in aggregate (clusters) [10, 13]. Woolhouse and colleagues suggest that this 20/80 rule may be useful for improving control of diseases such as malaria, which are transmitted heterogeneously and occur in clusters [14]. In other words, targeting the 20% source population could be more effective than targeting the whole population. Moreover, programs that fail to reach this clustered source population are less effective in reducing infection in the wider population [11, 14].

To facilitate targeted malaria control in high-risk populations [10, 11], understanding the epidemiological and spatiotemporal transmission of the disease is helpful. Malaria transmission is highly heterogeneous across geography and time due to complex interactions among parasites, vectors, and hosts [12, 15, 16]. The physical and seasonal environments directly influence spatial patterns of malaria transmission by creating nonrandom pathogen and vector distributions. Several studies have shown that mosquito distribution, prevalence, and incidence of malaria can vary over short distances between high-elevation and low-elevation areas, between neighboring villages, and even within a single village, due to small variations in risk factors [17–21]. For example, malaria is uncommon in high-elevation areas, because mosquitoes require high temperatures, high humidity, and suitable aquatic habitats to complete their pre-adult life cycles [22]. Conversely, areas with dams, irrigation canals, wetlands, man-made pools, rain pools, shoreline floods, and agricultural field puddles can influence the spatiotemporal pattern of malaria transmission [23, 24]. Transmission also is affected by proximity to mosquito breeding sites and the type of malaria control [19]. In the past decade, several studies have examined the spatiotemporal distribution of malaria in Ethiopia [16, 19, 25, 26]. However, these studies did not investigate how malaria interventions affect the heterogeneity of malaria transmission and the underlying risk factors for malaria clustering. Only one study tried to quantify the relationship between malaria transmission patterns and malaria intervention by assessing the use of insecticide-treated nets and indoor residual spraying (IRS) in a southern Ethiopian village with a high malaria infection rate [19]. Variation in malaria transmission according to different types of malaria control interventions (long-lasting insecticidal nets (LLINs) alone, IRS alone, a combination of LLINs and IRS) in areas of Ethiopia with low transmission rates has not yet been fully explored.

To fill this gap in the literature, we assessed the spatiotemporal patterns of malaria transmission in the presence of different malaria controls in a low-transmission area of southern-central Ethiopia. This study was a part of the cluster- randomized controlled trial utilizing the data collected for primary analysis published in elsewhere [27].We followed a large cohort of 34,548 people from October 2014 to January 2017 (121 weeks) in 13 *kebeles* (the lowest government administrative unit) that were targeted for the trial [27, 28]. The objectives of this study were to assess whether malaria infection were clustered in areas with LLINs alone, IRS alone, a combination of LLINs and IRS interventions, and to determine the risk factors for the observed clustering. The findings will help improve understanding of malaria distribution and prevention methods on a local scale.

## Materials and methods

### Ethical statement

The National Ethics Committee of the Ethiopian Ministry of Science and Technology (Ref: 3.10/446/06) and Institutional Review Board of the College of Health Sciences of Addis Ababa University approved the study protocol. We also obtained approval from the Regional Committee for Medical and Health Research Ethics, Western Norway (Ref: 2013/986/REK vest). Permission letters from the Oromia Regional State Health Bureau, East Shewa Zonal Health Department, and Adami Tullu District Health Office were written to the local administrators. Before implementing the study, a consultative meeting was conducted with representatives from each of these three organizations.

Sensitization meetings were conducted with the community elders and with *kebele* and village leaders to discuss the objectives, randomization procedures, implementation, follow-up, and expected outcomes of the study. Because most of the study population could not read and write, we obtained verbal informed consent from the heads of households or other household members older than 18 years. We used a standard information sheet to explain the purpose of the study. The participants were informed that their participation was voluntary and that they could refuse or withdraw from the study at any time. The participants were assured that refusal to participate in the study would not affect their right to use health services in the health posts. The information about the study was read to the study participants using an information sheet written in their language (Afan Oromo). Consent was recorded using a checkmark. As previously described, all participants who tested positive for *P*. *falciparum* or *P*. *vivax* on a rapid diagnostic test (RDT), a product of Premier Medical Corporation Limited, India, were treated at the health post with anti-malaria drugs according to national malaria treatment guidelines [6]. Individuals with severe illness were referred to the nearest health center for further investigation and treatment.

### Study area

**Fig 1:**
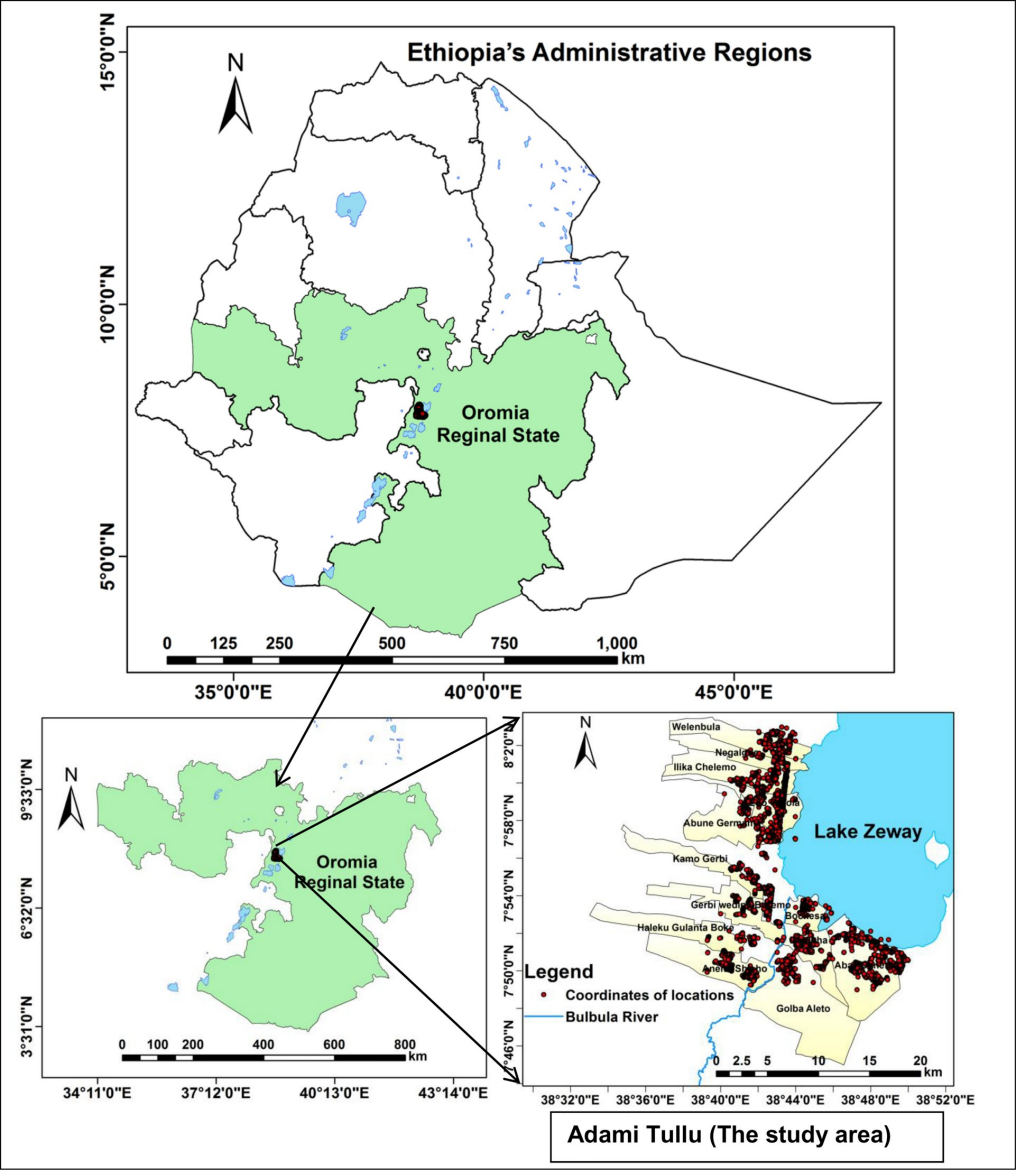
Map of Ethiopia, including the study location in the Adami Tullu district in southern-central Ethiopia. Red dots indicate households participating in the study.

The study was conducted in the Adami Tullu district of the Oromia Regional State, located approximately 160 km south of Addis Ababa, the capital city of Ethiopia (Fig 1). The district is in the Great Rift Valley, with altitudes ranging from 1500 m to 2300 m. The climate is semi-arid, with an average annual precipitation of 700 mm, which peaks during the rainy season in July and August. The annual rainfall of the district was 813 mm in 2014, 471 mm in 2015, and 890 mm in 2016. The average maximum temperature was 27°C in 2014, 29°C in 2015, and 28°C in 2016 [29]. The majority of the population lives in rural areas. Economic activity in the district is limited to subsistence farming, livestock rearing, and to a lesser extent, fishing in Lake Zeway. Houses consist of mud walls and thatched or corrugated iron roofs. The Oromo is the largest ethnic group in the district. Based on the 2007 national census, approximately 173,000 people lived in the district in 2014 [30]. The district has 48 *kebeles*, each with an average population of 1,000 to 5,000 people [30]. In 2014, there were two hospitals (one public and one non-governmental), nine public health centers, and 43 health posts in the district. Each *kebele* has at least one health post staffed by two health extension workers who report to the health center.

As a major health problem in the study area, malaria transmission is seasonal and unstable [31]. Most transmission occurs between September and December, following the monsoon rains in July and August [6]. A smaller peak of malaria transmission occurs between May and June, following rains in March and April [6]. Moreover, the shores and irrigated areas around Lake Zeway serve as potential mosquito breeding sites [23, 32]. The principal malaria vector in this area is *An*. *arabiensis*, and the two main malaria parasites are *P*. *falciparum* and *P*. *vivax* [33, 34]. During the study period, a severe drought occurred in the area following the El Nino effect in 2015 [35].

### Study design and participants

This study was part of a larger study, MalTrials, which aimed to evaluate whether the combined use of LLINs and IRS with propoxur provides additional protection against *P*. *falciparum* and *P*. *vivax* among all age groups, compared with LLINs alone or IRS alone [27, 28]. MalTrials was conducted in 13 *kebeles* adjacent to Lake Zeway. It used a 2x2, factorial, cluster-randomized, controlled design with four arms: LLIN + IRS; LLIN alone; IRS alone; and routine (control), which received standard Ethiopian malaria prevention. The unit of randomization was villages (clusters) that contained approximately 35 households and 196 people. The sample included 176 clusters within 5 km of Lake Zeway. In October 2014, eligible study participants received new PermaNet 2.0 LLINs free of charge. Based on national malaria guidelines [6], 7,740 LLINs were distributed to 3,006 households in the two eligible study arms (LLIN alone and LLIN + IRS). Eligible households (IRS alone and LLIN + IRS) received IRS with propoxur free of charge in September 2014, July 2015, and July 2016. See the MalTrials protocol and results for a detailed description of the study [27, 28].

This cohort study included all age groups and was conducted for 121 weeks, from October 2014 to January 2017. We recruited 24 field data collectors with college diplomas from the respective *kebeles* to conduct the baseline and update censuses and weekly follow-up data collection. Three supervisors were recruited to monitor the overall data collection process and data quality. All received five days of training on the use of questionnaires, interviewing techniques, household visits, and supervision. All study participants were followed on a weekly basis for the duration of the study period unless they were lost to follow-up (e.g., moved to another location, refused to participate, or died). Newcomers (individuals who joined a cohort as new household members) and children born during the study period also were included. The flow diagram illustrating the follow-up of study participants reported elsewhere [27]. Thirteen nurses (one nurse per health post per *kebele*) were recruited and trained on blood sample collection for the RDT, malaria diagnosis and treatment, and documentation of data. To ensure accurate data collection, refresher trainings were conducted in July 2015 and July 2016.

We assigned each household a metal plate with a unique identification number, and data collectors affixed the plate to the main entrance of the house. We also gave a unique identification card with a number corresponding to the unique number posted on the metal plate on the main entrance of each house. We advised the residents to come to the health posts with the unique identification card if they got febrile in the days between the weekly home visits. Study households were geo-referenced using a hand-held global positioning system (GPS) device (Garmin GPSMAP 60CSx, Garmin International Inc., Olathe, KS, USA).

### Baseline and subsequent censuses of the target population

We conducted a baseline census survey in July 2014 to collect individual- and household-level data. Individual-level data included age, sex, education status, religion, marital status, occupation, ethnicity, and morbidity. Household-level data included availability of household assets (e.g., television, radio, telephone, bed, chair, table, bike, animal cart, motor bike, car), access and types of latrine, source of drinking water, possession of land or animal, and type of construction material of the house. In July 2015 and July 2016, subsequent censuses were conducted to update for births, and in- and out-migration. To collect this information, the interviewer used a pre-tested questionnaire that was adopted from a pilot study of the trial [34]. The questionnaire was developed in English and then translated into the local language, Afan Oromo.

### Weekly follow-up data collection

Malaria episodes were identified using both active and passive case detection mechanisms. At weekly home visits, study participants with history of fever in the last 48 hours were registered and referred to a health post for malaria testing (active case detection). On days between weekly visits, the study participants were advised to report to the health post if they became febrile (passive case detection). At the weekly home visits, the names of the individuals who used the LLIN the night before the date of the visit were recorded.

Heads of households were the preferred respondent to all questions during data collection. In the absence of a head of household, family members ≥18 years old were asked to respond to questions. If no such person was available, the data collectors visited the house at least three more times within the same week.

### Malaria diagnosis and patient management

A malaria diagnosis was carried out at the health posts using a RDT. For the RDT, a nurse performed a single finger prick to collect a sample from the febrile patient and tested the sample. An individual with more than one positive RDT within a 30-day period was considered a single episode of malaria.

Based on the RDT results, patients with *P*. *falciparum* or mixed infection were treated with artemether-lumefantrine (Coartem), and patients with *P*. *vivax* infection were treated with chloroquine according to national malaria treatment guidelines [6]. Three health centers and one hospital were quarterly visited by field supervisors to collect data about malaria cases among study participants who visited the health facilities but did not report to our field workers. A malaria case was defined as a study participant who presented to the health post with symptoms of malaria (fever, chills, malaise, headache, or vomiting) and who had a positive RDT for *P*. *falciparum*, *P*. *vivax*, or mixed infection.

### Data analysis

Data were visualized using ESRI ArcMap 10.3.1 (ESRI, Redlands, CA, USA) software. The World Geodetic system 1984 and Universal Transverse Mercator Zone 37°N were used to define the coordinates’ projection. Three Microsoft Excel files (case, population, and coordinate) were prepared as input data for the Poisson probability model. Kulldorff’s spatial and space-time scan statistics were used to identify statistically significant retrospective clusters (purely spatial, purely temporal, and space-time) of high malaria rates using a Poisson probability model. SaTScan version 9.4.4 software was used to identify locations and periods of statistically significant clusters. The scan statistics computed data gradually across space and time to identify the number of observed and expected observations within each scanning window at each location and time. The scanning window shapes included a circle for space, an interval for time, and a cylinder with a circular base for space-time. In the space-time analysis, a circular geographic base represented space and corresponding height represented the time in months.

We used spatial scan statistics with circular windows of varying sizes from zero to a maximum radius of less than 50% of the total population at risk, allowing relocation across the study area. An unlimited number of overlapping circles of different sizes were obtained, and each circular window was a possible cluster. The corresponding log likelihood ratio (LLR) and relative risk (RR) were calculated for each circular window. The window with the maximum LLR was defined as the most likely cluster if the P-value <0.05. A criterion of “no geographic overlap” was used to report secondary clusters [36].

We applied space-time scan statistics using cylindrical windows with circular bases and heights corresponding to monthly timescale. The radius of each circular base allowed variation from zero to a maximum size of 50% of the total population, and the height of the cylinder varied in size from zero to 50% of the study period within one month. An infinite number of overlapping cylinders with different dimensions were obtained, and each cylinder was a candidate cluster. For each possible space-time cluster, the LLR and RR were calculated, and the most likely cluster was defined as the cylinder with the highest LLR having a P-value <0.05 [36]. The statistical significance of the clusters was tested using 999 Monte Carlo simulations. The P-value was obtained using a combination of the Monte Carlo, sequential Monte Carlo, and Gumbel approximations [36].

Spatial malaria clusters may appear due to underlying aggregation of one or more known risk factors within cluster areas. A non-random distribution of unstudied risk factors and spatial dependence could explain the lack of difference in known risk factors between a cluster and non-cluster area [37]. Tobler’s first law of geography on spatial dependency states that “everything is related to everything else, but nearby objects are more related than distant objects” [37]. Thus, to identify the underlining contributing factors for spatial malaria clustering observed in the study area, we compared malaria cases within identified spatial clusters (most likely and secondary) with malaria cases outside of the clusters. We applied a multilevel logistic regression model to account for malaria clustering effect within a group at the individual and village levels. Individual malaria cases (first level) were nested within the village (second level), assuming a difference in risk of spatial clustering of malaria between villages but a similar risk within a village.

Based on this assumption, the presence of clustering was checked before fitting the model. First, a null, single-level (standard), logistic regression model was fitted to the data. Then, a null, multilevel, logistic regression with the random village effect was fitted. The calculated likelihood ratio test statistics showed strong evidence of a village effect on the status of spatial clustering of malaria (Chi-square = 1024.50, P<0.001). Thus, to account for the clustering effect, we used a multilevel, logistic, regression model to estimate unadjusted and adjusted odds ratio (OR) with a 95% confidence interval (CI). The dependent variable is a binary variable and shows whether a malaria case was present within the identified spatial clusters or not (yes/no). We considered the following potential predictor variables based on their risk for malaria infection [13, 19, 34, 38–40]: age (<5, 5–14, 15–24 or >24 years), sex (male or female), family size (≤5 persons or >5 persons), educational status of head of household (illiterate, can read and write, primary, or secondary and above), occupational status of head of household (farmer or others), wealth index (poorest, poor, medium, rich or richest), intervention group (LLIN + IRS, LLIN only, IRS only or routine (control) arm), and distance from a lake or river (km) used as a continuous variable. Independent variables having P-values <0.25 in bivariate analyses were included in the multivariate logistic regression model for identifying independent risk factors of spatial malaria clustering, adjusting for other variables. Since the intervention group was our main variable that we wanted to test its effect on the final model, we included it in the multivariate logistic regression model irrespective of the P-value result in bivariate analysis. All tests were two-tailed, and the level of statistical significance was set at P<0.05.

We used principal component analysis (PCA) to construct a relative household wealth index [41, 42]. Fourteen household asset variables were included in the PCA model: presence of electricity and ownership of a television, radio, mobile telephone, chair, table, bed, bicycle, land, separate kitchen, livestock, livestock cart, types of roof (corrugated iron sheet vs. thatch) and wall (wood with mud/wood with mud and cement vs. no wall/only wood). The variables were dichotomized and coded as “1” if the household owned the asset or “0” if not. The Kaiser-Meyer-Olkin measure of sample adequacy was 0.79. A factor score derived from the first PCA was used to construct the wealth index. It represented 23.6% of the variance in the sample, with an Eigen value of 3.3. For descriptive purposes, the resulting index scores were used to assign households into quintiles: poorest, poor, medium, rich, and richest (see S1 File for the details).We used a proximity analysis tool in ESRI ArcMap 10.3.1 to calculate the distance (in km) between a household and the nearest potential vector breeding site from the border of Lake Zeway or the Bulbula River, and the nearest health facilities.

## Results

### Characteristics of the study population

The study comprised 34,548 people in 6,071 households. One-fifth, or 6,488 (18.8%), of the study participants were children younger than five years. Half, or 17,227 (50.2%), were male. More than half, or 3,345 (55.9%), of heads of households were illiterate, and 4,436 (74.5%) were farmers. Approximately half, or 3,106 (51.2%), of study households had a family size greater than five persons. One-third, or 2,051 (33.8), were located within 1 km of a potential mosquito breeding site. Table 1 describes the baseline study characteristics.

**Table 1:** Baseline characteristics of study participants and their households, southern-central Ethiopia, October 2014 to January 2017.

Variable	n (%)
**Age in years (n = 34548)**	
<5	6488 (18.8)
5–14	11136 (32.2)
15–24	6822 (19.8)
>24	10102 (29.2)
**Sex (n = 34548)**	
Male	17327 (50.2)
Female	17221 (49.8)
**Educational status of head of household (n = 5981)**^**a**^	
Illiterate	3345 (55.9)
Can read and write	560 (9.4)
Primary	1487 (24.9)
Secondary and above	589 (9.8)
**Occupational status of head of household (n = 5956)**^**a**^	
Farmer	4436 (74.5)
Others	1520 (25.5)
**Family size**^**a**^	
≤5 persons	2965 (48.8)
>5 persons	3106 (51.2)
**Wealth index**^**a**^	
Poorest	1216 (20.0)
Poor	1199 (19.8)
Medium	1229 (20.2)
Rich	1206 (19.9)
Richest	1221 (20.1)
**Intervention arm**^**a**^	
LLIN + IRS	1618 (26.7)
LLIN only	1388 (22.9)
IRS only	1527 (25.2)
Routine (control)	1538 (25.3)
**Distance from lake or river**^**a**^	
≤1 km	2051 (33.8)
>1 km	4020 (66.8)

^a^ Household-level characteristics (n = 6071 households, unless otherwise specified), LLIN = long-lasting insecticidal nets, IRS = indoor residual spraying

### Incidence of malaria

From October 1, 2014, to January 31, 2017, we documented 1,183 episodes of malaria in the study area. Of these, 652 (55.1%) were due to *P*. *falciparum* infection, 299 (25.3%) due to *P*. *vivax* infection, and 232 (19.6%) were mixed *P*. *falciparum* and *P*. *vivax* infections. Of the 34,548 people under follow-up during the 121 weeks, 1,059 (3.1%) developed at least one clinical episode of malaria with a range of 1 to 5 episodes. Similarly, of the 6,071 households, 812 (13.4%) had at least one malaria episode. Within the study period, the overall incidence of malaria was 16.5 episodes per 1,000 person-year observations (PYOs). These rates were 9.1 episodes per 1,000 PYOs for *P*. *falciparum*, 4.2 per 1,000 PYOs for *P*. *vivax*, and 3.2 per 1,000 PYOs for mixed infection. Table 2 shows the results.

**Table 2:** Malaria incidence rate per 1,000 person-year observations, southern-central Ethiopia, October 2014 to January 2017.

Variable	Person years	*Plasmodium falciparum*		*Plasmodium* *vivax*		Mixed		Total	
		Episodes	IR	Episodes	IR	Episodes	IR	Episodes	IR
**Total population**	71862	652	9.1	299	4.2	232	3.2	1183	16.5
**Age in years**									
<5	12742	150	11.8	69	5.4	51	4.0	270	21.2
5–14	23727	192	8.1	99	4.2	84	3.5	375	15.8
15–24	14000	69	4.9	47	3.4	32	2.3	148	10.6
>24	21393	241	11.3	84	3.9	65	3.0	390	18.2
**Sex**									
Male	36179	331	9.1	146	4.0	115	3.2	592	16.4
Female	35683	321	9.0	153	4.3	117	3.3	591	16.6
**Educational status of** **head of household**									
Illiterate	40028	333	8.3	165	4.1	112	2.8	610	15.2
Read and write	7396	80	10.8	44	5.9	47	6.4	171	23.1
Primary	17518	184	10.5	67	3.8	53	3.0	304	17.4
Secondary and above	5999	49	8.2	21	3.5	18	3.0	88	14.7
**Occupational status of** **head of household**									
Farmer	55156	499	9.0	256	4.6	199	3.6	954	17.3
Others	15434	146	9.5	39	2.5	31	2.0	216	14.0
**Family size**									
≤5 persons	21672	195	9.0	84	3.9	65	3.0	344	15.9
>5 persons	50190	457	9.1	215	4.3	167	3.3	839	16.7
**Wealth index**									
Poorest	14316	152	10.6	73	5.1	37	2.6	262	18.3
Poor	14406	153	10.6	61	4.2	42	2.9	256	17.8
Medium	14247	118	8.3	61	4.3	61	4.3	240	16.8
Rich	14390	115	8.0	52	3.6	55	3.8	222	15.4
Richest	14503	114	7.9	52	3.6	37	2.6	203	14.0
**Intervention arm**									
LLIN + IRS	18713	180	9.6	86	4.6	57	3.0	323	17.3
LLIN only	17244	173	10.0	69	4.0	36	2.1	278	16.1
IRS only	17153	153	8.9	68	4.0	68	4.0	289	16.8
Routine (control)	18752	146	7.8	76	4.1	71	3.8	293	15.6
**Distance from lake or river**									
≤1 km	22723	251	11.0	135	5.9	115	5.1	501	22.0
>1 km	49139	401	8.2	164	3.3	117	2.3	682	13.9

IR = Incidence rate, LLIN = long-lasting insecticidal nets, IRS = indoor residual spraying

### Spatial clustering of malaria

**Fig 2:**
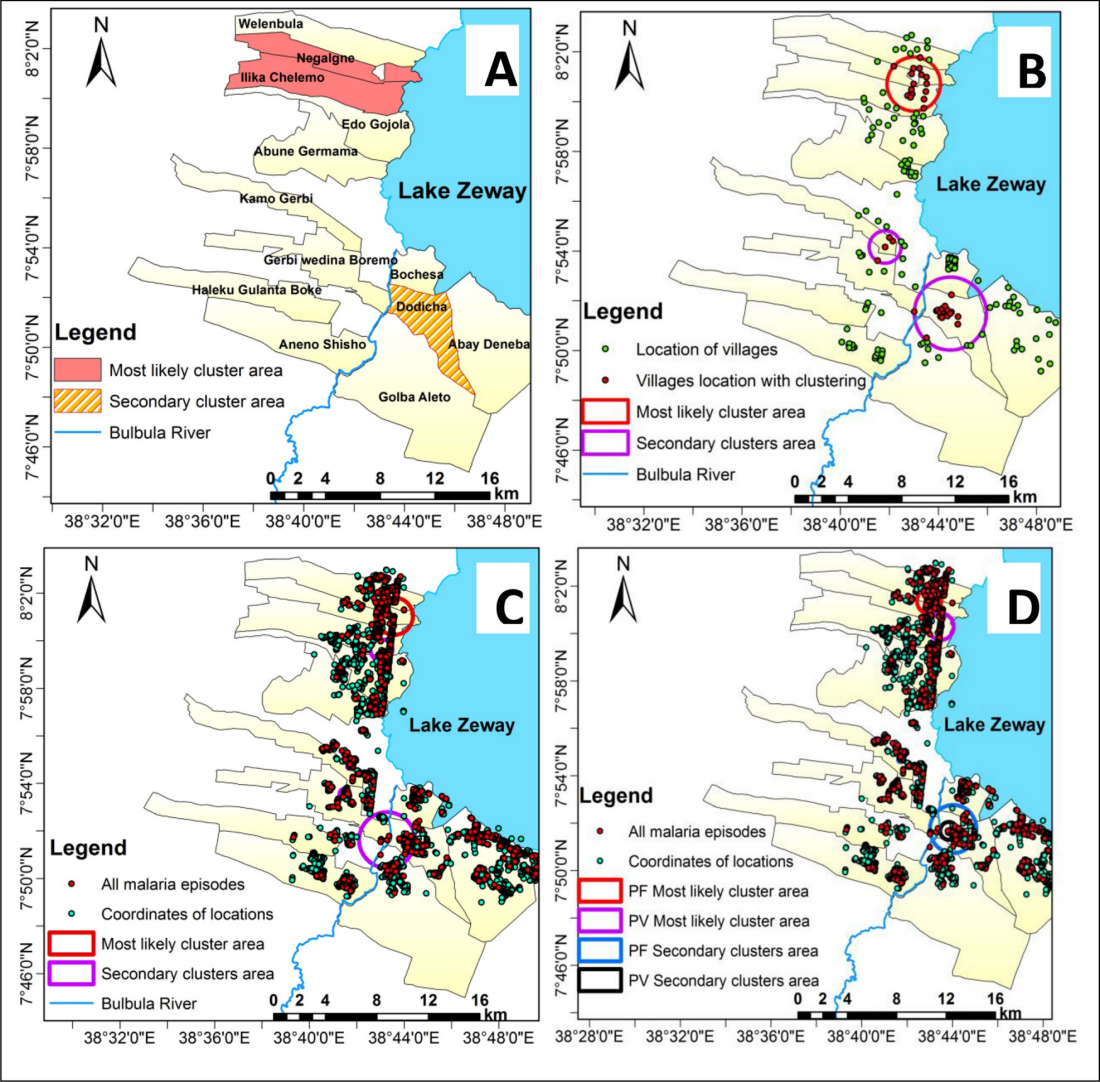
Most likely cluster and secondary clusters of all malaria types in southern-central Ethiopia at different scales using purely spatial scan statistics, October 2014 to January 2017. Panel A shows clustering at the *kebele* level, panel B at the village level, panel C at the household level, and panel D shows clustering of *Plasmodium falciparum* and *Plasmodium vivax* species at the household level.

We found areas with higher risk of malaria infection than in the underlying at-risk populations at the *kebele*, village, and household levels. The most likely and secondary significant spatial clusters for all malaria types (*P*. *falciparum*, *P*. *vivax*, or mixed) were identified at each geographic scale. The most likely cluster for each type occurred in the northern part of the study area, with the same geographic area at each geographic scale. The most likely clusters of *P*. *falciparum* and *P*. *vivax* did not overlapped geographically at household level. However, there was complete overlap in the secondary significant clusters of *P*. *falciparum* and *P*. *vivax* (Fig 2).

Moreover, a spatial clustering of malaria was detected among children 1 to 15 years and adults greater than 15 years in a separate analysis at household level. Despite variations in size of the clusters, all the identified significant clusters were overlapped among children 1 to 15 years and adults greater than 15 years (S1 Table and S1 Fig).

We conducted purely spatial scan analysis to identify areas with low rate of LLIN use using a discrete Poisson model. For this analysis, we used the average household level LLIN use both in LLIN alone and LLIN+IRS arms. Low LLIN use clusters were defined as areas having significantly lower average LLIN use than the underlining study area during the study period. Therefore, households or study participants in the study area were grouped into two categories: 1) households or study participants within low LLIN use clusters (clusters of significantly lower than expected LLIN use); and, 2) households or study participants in non-cluster (all other households or study participants outside the identified low LLIN use clusters). The analysis revealed the presence of significantly low LLIN use in the northern and southern parts of the study area. Meanwhile, the identified most likely high rate malaria cluster overlapped with the cluster of low rate of LLIN use (S2 Fig). Moreover, the risk of malaria infection in the identified low LLIN use clusters was significantly higher than non-cluster area by adjusting for distance from potential breeding site. People living in low LLIN use clusters were 2.20 times at increased risk of malaria infection than those living in non-cluster area (adjusted Hazard Ratio = 2.20, 95% CI = 1.80–2.60). See the S2 Table for details.

For all types of malaria episodes, the most likely significant cluster was identified in two of the 13 *kebeles* (Ilka Chalemo and Negalign), and a significant secondary cluster was detected in one *kebele* (Dodicha). Compared with people living in the other *kebeles*, those living in Ilka Chalemo and Negalign were 3.30 times more likely and those in Dodicha were 2.25 times more likely to contract malaria. This risk was 6.80 for *P*. *falciparum* in Negalign and 2.83 for *P*. *vivax* in Ilka Chalemo and Negalign. Table 3 shows the results.

**Table 3:** Purely spatial scan statistics of the most likely cluster and secondary clusters of malaria episodes at the *kebele* level, southern-central Ethiopia, October 2014 to January 2017.

Cluster	*Kebele*	Pop.	# episodes	Expected cases	Annual episodes per 1000	RR	LLR	P-value
All malaria types*								
Most likely	Ilka Chalemo, Negalign	3654	332	125.1	38.9	3.30	138.8	<0.001
Secondary	Dodicha	3360	231	115.1	29.4	2.25	51.6	<0.001
***Plasmodium falciparum***								
Most likely	Negalign	1132	122	21.4	46.1	6.80	120.4	<0.001
Secondary	Dodicha	3360	143	63.4	18.2	2.61	42.3	<0.001
Secondary	Qamo Garbi	1442	55	27.2	16.3	2.12	11.5	<0.001
***Plasmodium vivax***								
Most likely	Ilka Chalemo, Negalign	3654	75	31.6	8.8	2.83	25.1	<0.001
Secondary	Dodicha	3360	62	29.1	7.9	2.43	16.1	<0.001
Secondary	Garbi Widena	1617	26	14.0	6.9	1.94	4.4	0.047

* *Plasmodium falciparum*, *Plasmodium vivax*, or mixed, RR = Relative risk, LLR = Log likelihood ratio

People in villages within the most likely significant cluster area were 3.55 times more at risk of contracting all types of malaria than those living outside the cluster area. This risk was 8.69 for *P*. *falciparum* and 3.25 for *P*. *vivax* malaria infections. At the village level, each malaria type had two significant secondary clusters. Table 4 shows the results.

**Table 4:** Purely spatial scan statistics of the most likely cluster and secondary clusters of malaria episodes at the village level, southern-central Ethiopia, October 2014 to January 2017.

Cluster	# villages	Coordinates	Radius (km)	Pop.	# episodes	Expected cases	Annual episodes per 1000	RR	LLR	P-value
All malaria types*										
Most likely	17	8.012083 N, 38.716507 E	2.03	3605	346	123.44	41.05	3.55	159.3	<0.001
Secondary	19	7.858422 N, 38.741448 E	2.69	3055	246	104.60	34.30	2.69	77.7	<0.001
Secondary	4	7.902991 N, 38.697144 E	1.16	568	58	19.46	43.67	3.08	25.5	<0.001
***Plasmodium falciparum***										
Most likely	5	8.022632 N, 38.716322 E	0.95	927	126	17.49	58.13	8.69	150.1	<0.001
Secondary	11	7.863378 N, 38.737913 E	0.73	1637	103	30.89	26.91	3.77	56.28	<0.001
Secondary	6	7.920306 N, 38.692410 E	1.83	971	49	18.32	21.58	2.81	18.28	<0.001
Secondary	2	8.027165 N, 38.691838 E	1.28	246	17	4.64	29.55	3.73	9.83	0.004
***Plasmodium vivax***										
Most likely	18	8.006982 N, 38.724748 E	2.15	3602	82	31.17	9.74	3.25	33.63	<0.001
Secondary	1	7.893858 N, 38.692012 E	0.0	228	19	1.97	35.64	10.21	26.5	<0.001
Secondary	15	7.871003 N, 38.742309 E	1.83	2219	55	19.20	10.60	3.28	24.5	<0.001

* *Plasmodium falciparum*, *Plasmodium vivax*, or mixed, RR = Relative risk, LLR = Log likelihood ratio

Households within the most likely significant cluster were 4.75 times more at risk of contracting all types of malaria than households outside the cluster. This risk was 9.19 for *P*. *falciparum* and 5.79 for *P*. *vivax* malaria infection. At the household level, all malaria types had five secondary clusters, and the *P*. *falciparum* and *P*. *vivax* malaria species each had two secondary clusters. Table 5 shows the results.

**Table 5:** Purely spatial scan statistics of the most likely cluster and secondary clusters of malaria episodes at the household level, southern-central Ethiopia, October 2014 to January 2017.

Clusters	# locations	Coordinates	Radius (km)	Pop.	# episodes	Expected cases	Annual episodes per 1000	RR	LLR	P-value
All malaria types*										
Most likely	330	8.0175 N, 38.7262 E	1.5	1881	254	64.4	57.8	4.75	176.0	<0.001
Secondary	412	7.8606 N, 38.7213 E	2.2	2515	220	86.1	37.4	2.91	81.0	<0.001
Secondary	31	7.893 N, 38.6914 E	0.4	189	32	6.5	72.4	5.05	25.9	<0.001
Secondary	5	7.9122 N, 38.6949 E	0.2	26	10	0.9	164.5	11.32	15.1	<0.001
Secondary	123	7.9937 N, 38.7173 E	0.9	680	50	23.3	31.5	2.20	11.8	0.017
Secondary	28	7.954 N, 38.7132 E	0.2	225	24	7.7	45.6	3.16	11.1	0.027
***Plasmodium falciparum***										
Most likely	146	8.0232 N, 38.7161 E	1.0	828	120	15.6	62.0	9.19	149.3	<0.001
Secondary	443	7.8629 N, 38.7339 E	1.9	2716	136	51.3	21.4	3.09	54.2	<0.001
Secondary	7	7.9118 N, 38.6952 E	0.2	42	12	0.8	122.2	15.40	21.5	<0.001
***Plasmodium vivax***										
Most likely	156	8.0052 N, 38.7247 E	1.0	847	38	7.3	19.2	5.79	33.5	<0.001
Secondary	28	7.8927 N, 38.6914 E	0.4	174	19	1.5	46.7	13.41	31.2	<0.001
Secondary	187	7.8616 N, 38.7307 E	0.7	1174	40	10.2	14.6	4.39	26.6	<0.001

* *Plasmodium falciparum*, *Plasmodium vivax*, or mixed, RR = Relative risk, LLR = Log likelihood ratio

In a separate analysis for each study arm at the household level for all malaria types, all four study arms (LLIN + IRS, LLIN alone, IRS alone, and routine) had most likely clusters. Except for the LLIN + IRS arm, all other arms had two secondary clusters. Households within the most likely cluster in the LLIN + IRS arm were 4.54 times more at risk of contracting all types of malaria infections than households outside the cluster in the same intervention arm. This risk was 5.58 within the LLIN alone arm, 7.15 within the IRS alone arm, and 2.78 within the routine arm. See the S3 Table for details.

### Spatiotemporal clustering of malaria

**Fig 3:**
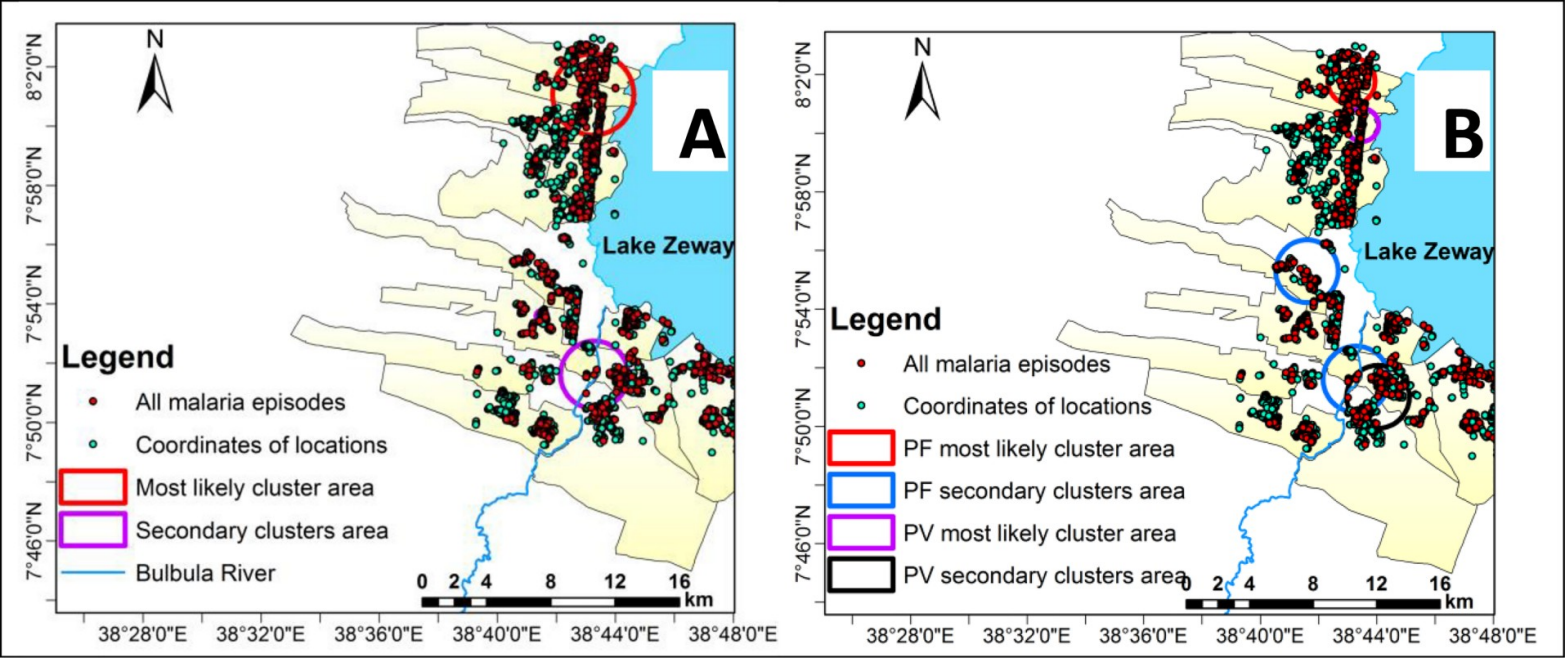
Most likely cluster and secondary clusters of malaria episodes identified using space-time scan statistics, southern-central Ethiopia, October 2014 to January 2017. Panel A shows all malaria episodes. Panel B shows *Plasmodium falciparum* and *Plasmodium vivax* episodes.

We analyzed space-time scan statistics at the household level. In the study district, both most likely and secondary spatiotemporal clusters were identified for *P*. *falciparum* and *P*. *vivax* infections. Each type had two secondary spatiotemporal clusters. Fig 3. Shows the identified most likely cluster and secondary clusters.

For all malaria types, the most likely spatiotemporal cluster lasted for 12 out of the 28-month study period, with varying start and end times, and clustering started on November 1, 2014. For *P*. *falciparum*, clustering began on December 1, 2014. For *P*. *vivax*, it began on October 1, 2014. The coverage area for all types of malaria (2.53 km) was larger than that for *P*. *falciparum* (1.49 km) and *P*. *vivax* (1.04 km). However, the relative risk of infection was highest for the *P*. *vivax* cluster, where people were 10.4 times more likely to contract *P*. *vivax* than households outside the cluster. This risk was 4.3 for all types of malaria and 8.9 for *P*. *falciparum*. See the S4 Table for details.

### Temporal clustering of malaria

In the study district, most likely purely temporal clusters were observed in all types of malaria, in *P*. *falciparum*, and in *P*. *vivax* malaria infections. The most likely purely temporal clusters were observed between September 1, 2015, and November 30, 2015, for all malaria types, when the risk of contraction in the purely temporal cluster was 2.25 times higher than during the rest of the study period. This risk was 2.36 for *P*. *falciparum* and 2.81 for *P*. *vivax*. Secondary purely temporal clusters were not observed in all categories of malaria infection in the study period. Table 6 and S3 Fig show the results.

**Table 6:** Purely temporal scan statistics of the most likely clusters of malaria, southern-central Ethiopia, October 2014 to January 2017.

Cluster	# locations	Timeframe	# episodes	Expected cases	Annual episodes per 1000	RR	LLR	P-value
All malaria types*	All	2015/9/1 to 2015/11/30	250	126	29.0	2.25	54.8	<0.001
***Plasmodium falciparum***	All	2015/9/1 to 2015/11/30	143	69.5	16.6	2.36	34.6	<0.001
***Plasmodium vivax***	All	2015/9/1 to 2015/11/30	75	31.9	8.7	2.81	24.8	<0.001

*Plasmodium falciparum, *Plasmodium vivax*, or mixed, RR = Relative risk, LLR = Log likelihood ratio

### Risk factors for spatial clustering of malaria

In this analysis, we compared the characteristics of malaria cases in the identified spatial clusters (n = 499) with characteristics of cases outside of the clusters (n = 560). In the bivariate, multilevel, logistic regression analysis, we found significant difference in cases within clusters and outside of clusters with regards to distance from a potential vector breeding site. Similarly, in the multivariate analysis, distance from a potential vector breeding site continued as significant predictor of spatial malaria clustering. Living 1 km closer to a potential vector breeding site increased the odds of being in a spatial cluster by 41.32 fold (adjusted OR = 41.32, 95% CI = 3.79–138.89). Meanwhile, we found no difference with regard to age, sex, family size, educational status of head of household, occupational status of head of household, wealth index, or study arm between malaria cases found in an identified spatial malaria clusters and cases outside of the clusters (Table 7).

**Table 7:** Multilevel, logistic regression for predictors of spatial clustering of all types of malaria at the household level, southern-central Ethiopia, October 2014 to January 2017.

Variables	Cases within identified spatial cluster		Unadjusted OR (95%CI)	P-value	Adjusted OR (95% CI)	P-value
	Yes n (%)	No n (%)				
**Age in years**						
<5	118 (48.2)	127 (51.8)	1		NA	
5–14	160 (47.9)	174 (52.1)	0.97 (0.40–2.34)	0.947		
15–24	63 (46.0)	74 (54.0)	0.18 (0.25–2.57)	0.718		
>24	158 (46.1)	185 (53.9)	1.62 (0.61–4.34)	0.332		
**Sex**						
Male	260 (49.4)	266 (50.6)	1		NA	
Female	239 (44.8)	294 (55.2)	1.17 (0.60–2.27)	0.664		
**Family size**						
≤5	149 (47.9)	162 (52.1)	1		NA	
>5	350 (46.8)	398 (53.2)	1.22 (0.59–2.51)	0.593		
**Educational status of head of household**						
No education	228 (41.5)	321 (58.5)	1		1	
Read and write	78 (52.3)	71 (47.7)	0.96 (0.28–3.38)	0.951	0.88 (0.10–7.57)	0.909
Primary	144 (52.9)	128 (47.1)	1.72 (0.77–3.84)	0.188	1.85 (0.76–4.54)	0.176
Secondary and above	47 (58.8)	33 (41.2)	2.88 (0.68–12.22)	0.152	3.45 (0.61–19.59)	0.162
**Occupational status of head of household**						
Farmer	397 (46.8)	452 (53.2)	1		NA	
Others	95 (47.7)	104 (52.3)	1.02 (0.75–2.33)	0.652		
**Wealth index**						
Poorest	93 (44.0)	109 (54.0)	1		1	
Poor	106 (46.3)	123 (53.7)	0.67 (0.24–1.85)	0.441	1.70 (0.47–6.15)	0.421
Medium	89 (42.4)	121 (57.6)	0.41 (0.09–1.85)	0.247	0.70 (0.18–2.71)	0.604
Rich	117 (52.9)	104 (47.1)	1.04 (0.19–5.79)	0.966	1.69 (0.14–20.33)	0.680
Richest	94 (47.7)	103 (52.3)	1.18 (0.24–5.86)	0.841	1.67 (0.16–17.59)	0.668
**Intervention arm**						
LLIN + IRS	136 (47.2)	152 (52.8)	1		1	
LLIN only	112 (44.3)	141 (55.7)	0.35 (0.01–9.22)	0.533	0.56 (0.23–1.38)	0.208
IRS only	123 (47.1)	138 (52.9)	0.33 (0.01–8.58)	0.508	0.45 (0.16–1.26)	0.130
Routine (control)	128 (49.8)	129 (50.2)	0.41 (0.02–8.58)	0.563	1.32 (0.48–3.62)	0.595
Distance from lake or river (km)*						
Mean (SD)	1.30 (1.02)	1.88 (1.38)	33.67 (10.69–106.04)^¥^	<0.001	41.32 (3.79–138.89)^¥^	<0.001

n = number of malaria cases, OR = Odds ratio, NA = not applicable (P>0.25 in bivariate analysis), LLIN = long-lasting insecticidal nets, IRS = indoor residual spraying

*At village level: mean (SD) distance from potential breeding site for clusters = 1.40 (0.90), for non-clusters = 2.10 (1.51), unadjusted OR (95%CI) = 1.52 (1.11–2.04).

^¥^The reciprocal of the OR (95% CI) is presented to show the risk of proximity to a potential vector breeding site.

To identify village level risk factor for spatial clustering of malaria, we used logistic regression model. The three independent variables included in the village level analysis were: The intervention arm, distance from the nearest health facilities and distance from the potential vector breeding site. The only variable that was significantly associated with spatial clustering of malaria was distance from the potential vector breeding site. Villages found in 1 km closer to a potential vector breeding site at increased odds of being in a spatial cluster by 1.5 fold (adjusted OR = 1.5, 95% CI = 1.15–1.93). See the S5 Table for details.

## Discussion

We found purely spatial, purely temporal, and spatiotemporal clustering of malaria infection in southern-central Ethiopia. This finding shows that malaria infection was not randomly distributed at the *kebele*, village, or household levels in areas with different malaria control interventions.

As part of a large, cluster-randomized control trial, our study compared the incidence of malaria transmission based on combined interventions (LLINs and IRS) and individual interventions (LLINs alone or IRS alone) [27, 28]. We followed a large cohort of people (n = 34548) in the rural communities of the Adami Tullu district from October 2014 to January 2017 (28 months) to evaluate malaria risk in low-risk and high-risk malaria transmission seasons. The study findings could improve understanding of the micro-geographic heterogeneity of malaria transmission, which can be useful for planning targeted malaria control interventions in small areas. Moreover, the findings can be generalized to many parts of Ethiopia with similar geographic, topographic, and socio-economic conditions.

In the current study, the overall malaria incidence was 16.5 episodes per 1,000 PYOs over the 28 months of follow-up. The incidence was lower than that found in a pilot study that was conducted in the same study area from August 2013 to December 2013, in which the average incidence was 4.6 episodes per 10,000 person-week observations (approximately 24 episodes per 1,000 PYOs) [34]. The difference may be due to the timing of the pilot study, which was conducted during the high malaria transmission season. The incidence also was lower than that of a previous longitudinal study from southern Ethiopia (45.1 per 1,000 PYOs) [19] and the national average incidence between 2011 and 2016 (29.0 cases per 1,000 PYOs) [3]. This lower incidence of malaria observed in the current study area could be related to climate irregularity caused by the 2015 El Nino effect [35] or to differences in coverage of malaria control interventions.

Using spatial scan statistics, we identified locations with high risks of malaria infection. Similar findings have been reported elsewhere in Ethiopia [19, 25, 26, 43]. In the present study, three *kebeles* out of 13 accounted for nearly half (47.6%) of all malaria episodes, and 15.3% of households in the identified clusters accounted for half (50%) of all malaria episodes. Thus, malaria infection was localized and frequent in high-burden clusters in low malaria transmission settings. Targeted interventions in these high-burden clusters can optimize resources and improve effectiveness of malaria elimination programs [10, 11].

Despite variations in size and location of spatial clustering of malaria between study groups, all four study arms (LLINs + IRS, LLIN alone, IRS alone, and routine) showed malaria clustering in separate analyses, with no significant differences in the risk of clustering at individual case or village level (Table 7, S5 Table). The results from the main trials also showed no significant differences in the incidence of malaria across study arms [27]. These results indicate that using LLINs and IRS in combination or alone may not prevent malaria clustering in areas with low rates of malaria transmission. The reason for this lack of difference might be related to the effect of residual transmission, which primarily occurs due to the outdoor and early evening indoor biting behavior of *An*. *arabiensis* in the study area [27, 44, 45]. In contrast to our study, another cohort study in southern Ethiopia shows that the use of IRS with deltamethrin affected the spatiotemporal clustering of malaria, but LLINs did not [19]. This difference in findings might be due to the difference in malaria burdens between the study areas (16.5 episodes per 1,000 PYO in our study vs. 45.1 episodes per 1,000 PYO in the other study) [19].

The space-time scan statistics identified high-risk areas for all malaria types over space and time. All the most likely clusters were in locations with identified spatial clusters. Although the overall incidence of malaria was low in the study area and period, there were relatively high malaria infections in 12 of 28 months from November 2014 to November 2015. These spikes in infection rates may be related to warmer temperatures from the El Nino effect in 2015 [29]. As the warmest year on record, 2015 had an average maximum temperature of 29°C, which was 2°C warmer than 2014 and 1°C warmer than 2016 [29]. This warmer temperature may facilitate quick sporogonic development of *Plasmodium* species [46]

The purely temporal cluster analysis aimed to identify high-risk periods for malaria transmission. A significant temporal cluster was observed from September 1, 2015, to November 30, 2015, with peaks in October. This high-risk period is consistent with the high malaria transmission season that occurs in most parts of Ethiopia, following heavy rains in June, July, and August [6, 16]. Thus, malaria interventions before September might further reduce malaria transmission in the study area.

The duration and peaks of infection varied in the study period. For example, in 2015, two major peaks of malaria episodes were observed in January and October. In 2016, two major peaks occurred in June and September. Smaller peaks occurred in between the major peaks each year. In addition to the major risk factors for malaria infection, such as rainfall, temperature, and relative humidity [47–49], local irrigation activity in the study area also may have influenced the observed smaller peaks of malaria infection in dry seasons [23, 32].

We compared cases identified within spatial clusters and those outside of the clusters to further understand the risk factors for malaria clustering. In this analysis, the only factor independently associated with malaria clustering was living close to a potential vector breeding site. The proximity to Lake Zeway or the Bulbula River, which have the most confirmed breeding sites [23, 50], increased the risk of malaria clustering at individual and village level analysis. Previous studies also have reported that close proximity to these sites increases the risk of malaria infection and clustering [19, 48, 51–54]. It is not a surprise to see higher risk of infection in a locality near breeding site of potentially infective Anopheles mosquitoes [13]. Therefore, targeting the households or villages found closer to potential vector breeding site with effective malaria control measures could further decrease the burden of malaria infection. Moreover, there was an indication that clustering of malaria associated with low LLIN use, because the most likely cluster of malaria was imbedded within the cluster of low rate of LLIN use, and also there was increased risk of malaria infection in low LLIN use clusters. Thus, it needs to ensure the utilization of LLINs after distribution by all households to maximize the effect of LLINs on malaria infection.

The Ethiopian Ministry of Health plans to eliminate malaria in 2020 in selected districts with low malaria transmission [55]. To achieve this plan, the Ministry may consider targeted intervention at the *kebele*, village, or individual household level in areas with high-burden malaria clusters. Ideally, such targeted intervention strategies will optimize resources and increase program coverage and effectiveness [11]. To ensure effective implementation of these intervention mechanisms, the Ministry might consider improving identification of malaria clusters.

We believe that our study has some limitations. First, comparing malaria clustering by intervention arm might have been affected by the context of our study period, during which unexpectedly dry and warmer weather conditions occurred following the El Nino effect in 2015. Annual rainfall declined by 60%, and the average temperature increased by 2 ^o^C above normal [29]. Severe drought and food shortage also occurred in the study area [35]. Due to the unexpected weather conditions and other behaviors [56], ownership and use of LLINs in the study period dramatically declined after six months of intervention [57, 58]. Our study results may have been different if LLIN ownership and use were higher. Second, a spill-over effect could have occurred between villages of each intervention arm, which may have diluted any difference in the clustering of malaria. Third, we used RDT to confirm the diagnosis of malaria. However, RDT is less sensitive in detecting submicroscopic infection than Polymerase Chain Reaction (PCR) [59, 60]. Compared to all infection, the proportion low density malaria parasite infection is common and have been estimated to be about 20–50% of all malaria episodes in low transmission setting [61, 62]. Therefore, a considerable proportion of submicroscopic infection might be missed in the current study. A study shows that malaria hotspots identified by RDT were not predictive of PCR or microscopy, and long-term stability of hotspots was not observed by RDT in low malaria transmission setting [63]. Moreover, we cannot rule-out the presence of other plasmodium species (such as *Plasmodium ovale* and *Plasmodium malariae)*. However, the prevalence of these infections is less than 1% of malaria cases [64]. Fourth, we opted to use a circular window in the spatial scan statistics to identify the clusters due to its ability to detect other cluster shapes and isotropy with respect to map rotation; however, the true clusters may be elliptic or rectangular. Scan statistics using elliptic or rectangular windows cannot detect these shapes, though, unless all possible angles are considered, which is difficult to compute [36]. Fifth, we did not include all possible risk factors for malaria clustering, such as irrigation-related vector breeding sites and climate (rainfall, temperature, relative humidity). The non-random distribution of these excluded risk factors could be responsible for the observed clustering of malaria.

## Conclusion

In conclusion, the risk of malaria infection varied significantly in the study area. We observed high rates of spatial, temporal, and spatiotemporal clustering of malaria episodes at the *kebele*, village, and household levels. Spatial clustering occurred in all four study arms, and the risk of clustering was similar across the arms. Therefore, the results of this study can be used in planning and implementation of malaria control strategies at micro-geographic scale.

## Supporting information

S1 Table Purely spatial scan statistics of the most likely cluster and secondary clusters of malaria episodes at the household level among children and adults, southern-central Ethiopia, October 2014 to January 2017. (DOCX)

S2 Table The risk of malaria in the low long-lasting insecticidal net use clusters and non-clusters, southern-central Ethiopia, October 2014 to January 2017. (DOCX)

S3 Table Purely spatial scan statistics of the most likely cluster and secondary clusters of all types of malaria episodes by intervention arm at individual level, southern-central Ethiopia, October 2014 to January 2017. (DOCX)

S4 Table Space-time scan statistics of the most likely cluster and secondary clusters of malaria at the household level, southern-central Ethiopia, October 2014 to January 2017. (DOCX)

S5 Table Predictors of spatial clustering of all types of malaria at the village level, southern-central Ethiopia, October 2014 to January 2017. (DOCX)

S1 Fig The most likely cluster and secondary clusters of malaria episodes at the household level among children and adults, southern-central Ethiopia, October 2014 to January 2017. (TIF)

S2 Fig Most likely and secondary clusters of all malaria types and areas with low long- lasting insecticidal net use in southern-central Ethiopia, October 2014 to January 2017. (TIF)

S3 Fig Monthly malaria incidence showing temporal clusters of all types of malaria (shaded part) and total rainfall (lagged by one month), southern-central Ethiopia, October 2014 to January 2017. (TIF)

S1 File Variables used to construct the wealth index and their correlation with the first component. (DOCX)
