# Supplementary material for: Spatiotemporal clustering of malaria in southern-central Ethiopia: A community-based cohort study
Source: PLoS One. 2019 Sep 30;14(9):e0222986. doi: 10.1371/journal.pone.0222986 (PMC6768540; doi:10.1371/journal.pone.0222986)
Supplement: S1 Table — (DOCX) [file pone.0222986.s001.docx]

| **Types of malaria** | **Age (years)** | **Clusters** | **# locations** | **Pop.** | **# episodes** | **Expected cases** | **Annual episodes per 1000** | **RR** | **LLR** | **P-value** |
| --- | --- | --- | --- | --- | --- | --- | --- | --- | --- | --- |
| **All malaria types*** | **1-15** | Most likely | 762 | 2427 | 236 | 88.67 | 41.59 | 3.7 | 196.3 | <0.001 |
|  |  | Secondary | 305 | 1114 | 116 | 40.70 | 44.53 | 3.3 | 51.3 | <0.001 |
|  |  | Secondary | 23 | 83 | 19 | 3.03 | 97.90 | 6.4 | 19.1 | <0.001 |
|  | **>15** | Most likely | 342 | 937 | 112 | 30.19 | 51.12 | 4.46 | 72.4 | <0.001 |
|  |  | Secondary | 58 | 149 | 31 | 4.80 | 88.98 | 6.81 | 32.3 | <0.001 |
|  |  | Secondary | 92 | 257 | 25 | 8.28 | 41.60 | 3.12 | 11.2 | <0.001 |
| ***Plasmodium falciparum*** | **1-15** | Most likely | 303 | 1008 | 94 | 19.21 | 39,88 | 6.5 | 84.5 | <0.001 |
|  |  | Secondary | 393 | 1477 | 76 | 28.15 | 22.0 | 3.2 | 31.7 | <0.001 |
|  |  | Secondary | 5 | 22 | 7 | 0.42 | 136.1 | 17.0 | 13.2 | 0.005 |
|  | **>15** | Most likely | 146 | 387 | 53 | 7.29 | 58.57 | 8.61 | 63.2 | <0.001 |
|  |  | Secondary | 58 | 149 | 23 | 2.81 | 66.01 | 8.79 | 28.9 | <0.001 |
|  |  | Secondary | 126 | 341 | 23 | 6.42 | 28.84 | 3.80 | 13.2 | <0.001 |
| ***Plasmodium vivax*** | **1-15** | Most likely | 239 | 748 | 32 | 7.35 | 18.29 | 5.2 | 24.4 | <0.001 |
|  |  | Secondary | 18 | 65 | 12 | 0.64 | 78.95 | 20.2 | 24.2 | <0.001 |
|  |  | Secondary | 344 | 1289 | 38 | 12.66 | 12.60 | 3.6 | 18.6 | <0.001 |
|  | **>15** | Most likely | 26 | 59 | 7 | 0.45 | 50.74 | 16.6 | 12.9 | 0.0046 |
|  |  | Secondary | 152 | 403 | 15 | 3.05 | 15.91 | 5.5 | 12.6 | 0.0062 |
|  |  | Secondary | 66 | 223 | 11 | 1.69 | 21.09 | 7.07 | 11.7 | 0.024 |

**S1 Table. Purely spatial scan statistics of the most likely cluster and secondary clusters of malaria episodes at the household level among children and adults, southern-central Ethiopia, October 2014 to January 2017**
