# Supplementary material for: Spatiotemporal clustering of malaria in southern-central Ethiopia: A community-based cohort study
Source: PLoS One. 2019 Sep 30;14(9):e0222986. doi: 10.1371/journal.pone.0222986 (PMC6768540; doi:10.1371/journal.pone.0222986)
Supplement: S2 Table — (DOCX) [file pone.0222986.s002.docx]

| **Variable** | **Person**  **years** | ***Plasmodium***  ***falciparum*** | | ***Plasmodium  vivax*** | | **Mixed** | | **All types of malaria** | | **Crude**  **HR (95% CI)** | **Adjusted**  **HR (95% CI)*** |
| --- | --- | --- | --- | --- | --- | --- | --- | --- | --- | --- | --- |
|  |  | **Episodes** | **IR (95% CI)** | **Episodes** | **IR (95% CI)** | **Episodes** | **IR (95% CI)** | **Episodes** | **IR (95% CI)** |  |  |
| **Significant Low rate LLIN use cluster** |  |  |  |  |  |  |  |  |  |  |  |
| **No** | 16706 | 103 | 6.2 (5.1-7.4) | 50 | 3.0 (2.2-3.9) | 28 | 1.7 (1.1-2.4) | 181 | 10.8 (9.3-12.5) | 1 | 1 |
| **Yes** | 19250 | 250 | 13.0 (11.4-14.6) | 105 | 5.5 (4.5-6.6) | 65 | 3.4 (2.6-4.3) | 420 | 21.8 (19.8-24.0) | 1.98 (1.54-2.55) | 2.20 (1.80-2.60) |

**S2 Table. Malaria incidence in the low rate long-lasting insecticidal net use clusters and non-clusters, southern-central Ethiopia, October 2014 to January 2017**

IR=Incidence rate (All IR was calculated per 1,000 person-year observations), LLIN= long-lasting insecticidal nets, HR=hazard ratio,

*adjusted HR calculated for all types of malaria episodes and adjusted for distance from potential mosquitoes breeding site
