# Supplementary material for: Spatiotemporal clustering of malaria in southern-central Ethiopia: A community-based cohort study
Source: PLoS One. 2019 Sep 30;14(9):e0222986. doi: 10.1371/journal.pone.0222986 (PMC6768540; doi:10.1371/journal.pone.0222986)
Supplement: S3 Table — (DOCX) [file pone.0222986.s003.docx]

**S3 Table. Purely spatial scan statistics of the most likely cluster and secondary clusters of all types of malaria episodes by intervention arm at individual level, southern-central Ethiopia, October 2014 to January 2017**

| **Clusters** | **# locations** | **Pop.** | **# episodes** | **Expected cases** | **Annual episodes per 1000** | **RR** | **LLR** | **P-value** |
| --- | --- | --- | --- | --- | --- | --- | --- | --- |
| **Long-lasting insecticidal nets +**  **Indoor residual spraying arm** |  |  |  |  |  |  |  |  |
| Most likely | 305 | 1780 | 169 | 63.0 | 40.6 | 4.54 | 86.0 | <0.001 |
| **Long-lasting insecticidal nets only arm** | |  |  |  |  |  |  |  |
| Most likely | 103 | 617 | 88 | 21.3 | 61.0 | 5.58 | 67.6 | <0.001 |
| Secondary | 19 | 118 | 24 | 4.1 | 87.0 | 6.35 | 23.4 | <0.001 |
| Secondary | 9 | 53 | 11 | 1.83 | 88.8 | 6.21 | 10.7 | 0.011 |
| **Indoor residual spraying only arm** | |  |  |  |  |  |  |  |
| Most likely | 50 | 291 | 58 | 9.8 | 85.2 | 7.15 | 59.3 | <0.001 |
| Secondary | 26 | 137 | 38 | 4.6 | 118.6 | 9.32 | 48.7 | <0.001 |
| Secondary | 5 | 26 | 10 | 0.9 | 164.5 | 11.78 | 15.4 | <0.001 |
| **Routine (control) arm** |  |  |  |  |  |  |  |  |
| Most likely | 377 | 2335 | 147 | 77.8 | 26.9 | 2.78 | 36.9 | <0.001 |
| Secondary | 71 | 384 | 45 | 12.8 | 50.1 | 3.97 | 26.3 | <0.001 |
| Secondary | 16 | 103 | 15 | 3.4 | 62.3 | 4.55 | 10.8 | 0.009 |

RR=Relative risk, LLR=Log likelihood ratio
