# Supplementary material for: Spatiotemporal clustering of malaria in southern-central Ethiopia: A community-based cohort study
Source: PLoS One. 2019 Sep 30;14(9):e0222986. doi: 10.1371/journal.pone.0222986 (PMC6768540; doi:10.1371/journal.pone.0222986)
Supplement: S4 Table — (DOCX) [file pone.0222986.s004.docx]

**S4 Table. Space-time scan statistics of the most likely cluster and secondary clusters of malaria at the household level, southern-central Ethiopia, October 2014 to January 2017**

| **Cluster** | **# locations** | **Timeframe** | **Pop.** | **# episodes** | **Expected cases** | **Annual episodes per 1000** | **RR** | **LLR** | **P-value** |
| --- | --- | --- | --- | --- | --- | --- | --- | --- | --- |
| **All malaria**  **types*** | | | | | | | | | |
| Most likely | 842 | Nov. 2014 to Nov. 2015 | 4706 | 267 | 74.5 | 52.5 | 4.31 | 166.0 | <0.001 |
| Secondary | 403 | Sep. 2015 to Sep. 2016 | 2377 | 142 | 37.7 | 55.1 | 4.12 | 88.8 | <0.001 |
| Secondary | 29 | Oct. 2014 to Jan. 2015 | 183 | 18 | 0.9 | 292.1 | 20.22 | 39.9 | <0.001 |
| ***Plasmodium***  ***falciparum*** | | | | | | | | | |
| Most likely | 355 | Dec. 2014 to Dec. 2015 | 1989 | 128 | 17.4 | 59.4 | 8.89 | 155.1 | <0.001 |
| Secondary | 398 | Sep. 2015 to Sep. 2016 | 2340 | 77 | 20.5 | 30.4 | 4.07 | 48.1 | <0.001 |
| Secondary | 145 | May. 2015 to Jun. 2016 | 899 | 34 | 8.5 | 32.4 | 4.98 | 22.2 | <0.001 |
| ***Plasmodium***  ***vivax*** | | | | | | | | | |
| Most likely | 163 | Oct. 2014 to Oct. 2015 | 847 | 32 | 3.4 | 34.8 | 10.37 | 44.6 | <0.001 |
| Secondary | 651 | Oct. 2015 to Nov. 2015 | 3826 | 26 | 2.4 | 40.7 | 11.89 | 39.7 | <0.001 |
| Secondary | 28 | Oct. 2014 to Jan. 2015 | 174 | 12 | 0.2 | 204.8 | 57.58 | 36.6 | <0.001 |

* *Plasmodium falciparum*, *Plasmodium vivax*, or mixed, RR=Relative risk, LLR=Log likelihood ratio
