# Supplementary material for: Spatiotemporal clustering of malaria in southern-central Ethiopia: A community-based cohort study
Source: PLoS One. 2019 Sep 30;14(9):e0222986. doi: 10.1371/journal.pone.0222986 (PMC6768540; doi:10.1371/journal.pone.0222986)
Supplement: S5 Table — (DOCX) [file pone.0222986.s005.docx]

**S5 Table. Predictors of spatial clustering of all types of malaria at the village level, southern-central Ethiopia, October 2014 to January 2017**

| **Variables** | **Village within identified spatial cluster** | | **Unadjusted**  **OR (95%CI)** | **P-value** | **Adjusted**  **OR (95% CI)** | **P-value** |
| --- | --- | --- | --- | --- | --- | --- |
|  | **Yes**  **n (%)** | **No**  **n (%)** |  |  |  |  |
| **Intervention arm** | | | | | | |
| LLIN + IRS | 13 (29.5) | 31 (70.5) | 1 |  |  |  |
| LLIN only | 11 (25.0) | 33 (75.0) | 0.79 (0.31-2.04) | 0.633 | 0.88 (0.33-2.38) | 0.802 |
| IRS only | 7 (15.9) | 37 (84.1) | 0.45 (0.16-1.27) | 0.133 | 0.50 (0.17-1.50) | 0.218 |
| Routine (control) | 8 (18.2) | 36 (81.8) | 0.53 (0.19-1.45) | 0.216 | 0.67 (0.23-1.93) | 0.463 |
| **Distance from nearest**  **health facility (km)** | | | | | | |
| Mean (SD) | 1.75 (0.67) | 2.01 (0.88) | 0.68 (0.45-1.02) | 0.062 | 0.68 (0.42-1.08) | 0.102 |
| **Distance from**  **lake or river (km)** | | | | | | |
| Mean (SD) | 1.40 (0.90) | 2.10 (1.51) | 1.51 (1.17-1.93)^¥^ | 0.001 | 1.50 (1.15-1.93)^¥^ | 0.003 |

n=number of village, OR=Odds ratio, LLIN= long-lasting insecticidal nets, IRS= indoor residual spraying

^¥^The reciprocal of the OR (95% CI) is presented to show the risk of proximity to a potential vector breeding site.
