# Supplementary figures and images for: Spatiotemporal clustering of malaria in southern-central Ethiopia: A community-based cohort study
Source: PLoS One. 2019 Sep 30;14(9):e0222986. doi: 10.1371/journal.pone.0222986 (PMC6768540; doi:10.1371/journal.pone.0222986)

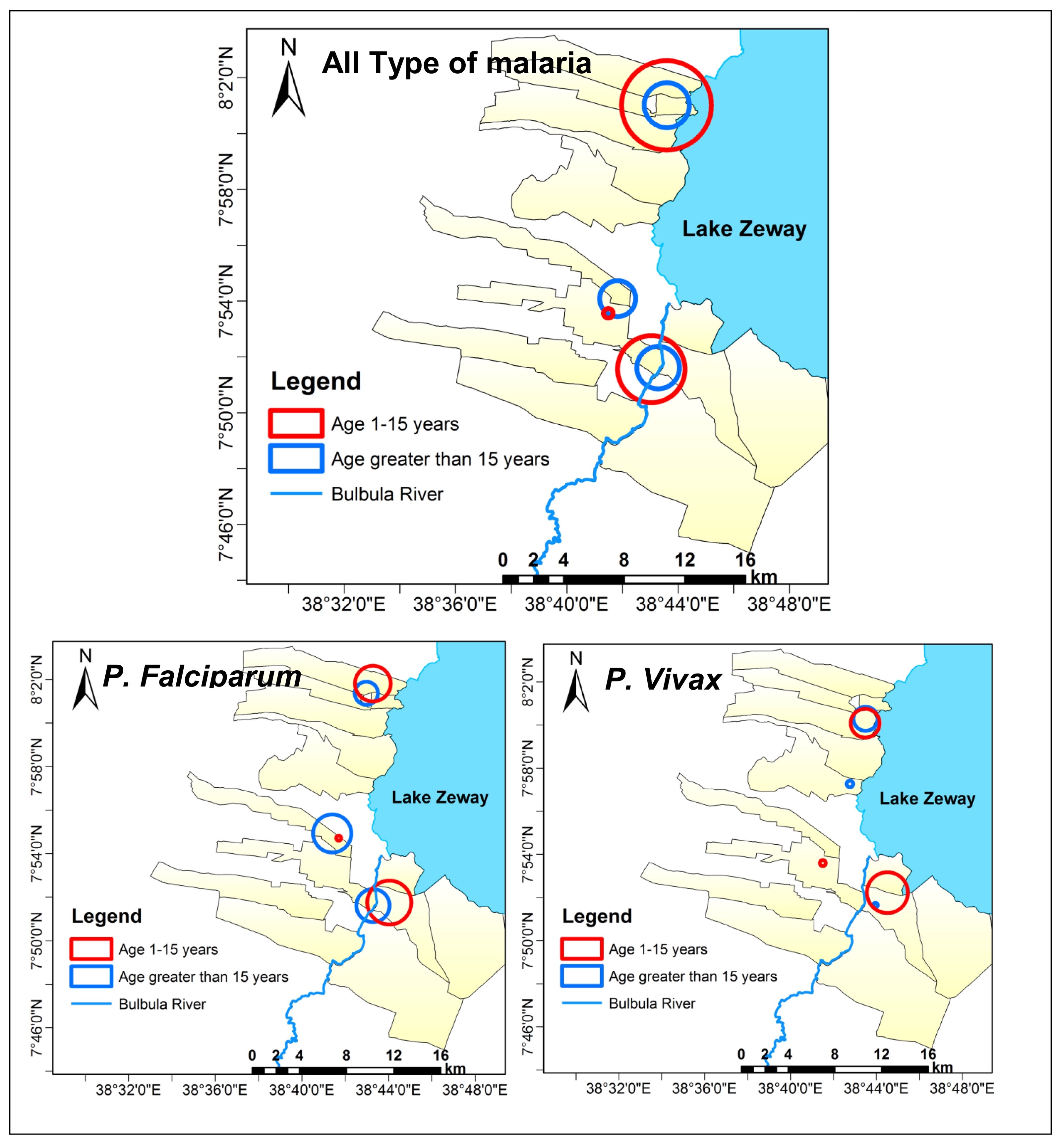

Supplement: S1 Fig — (TIF) [file pone.0222986.s006.tif]

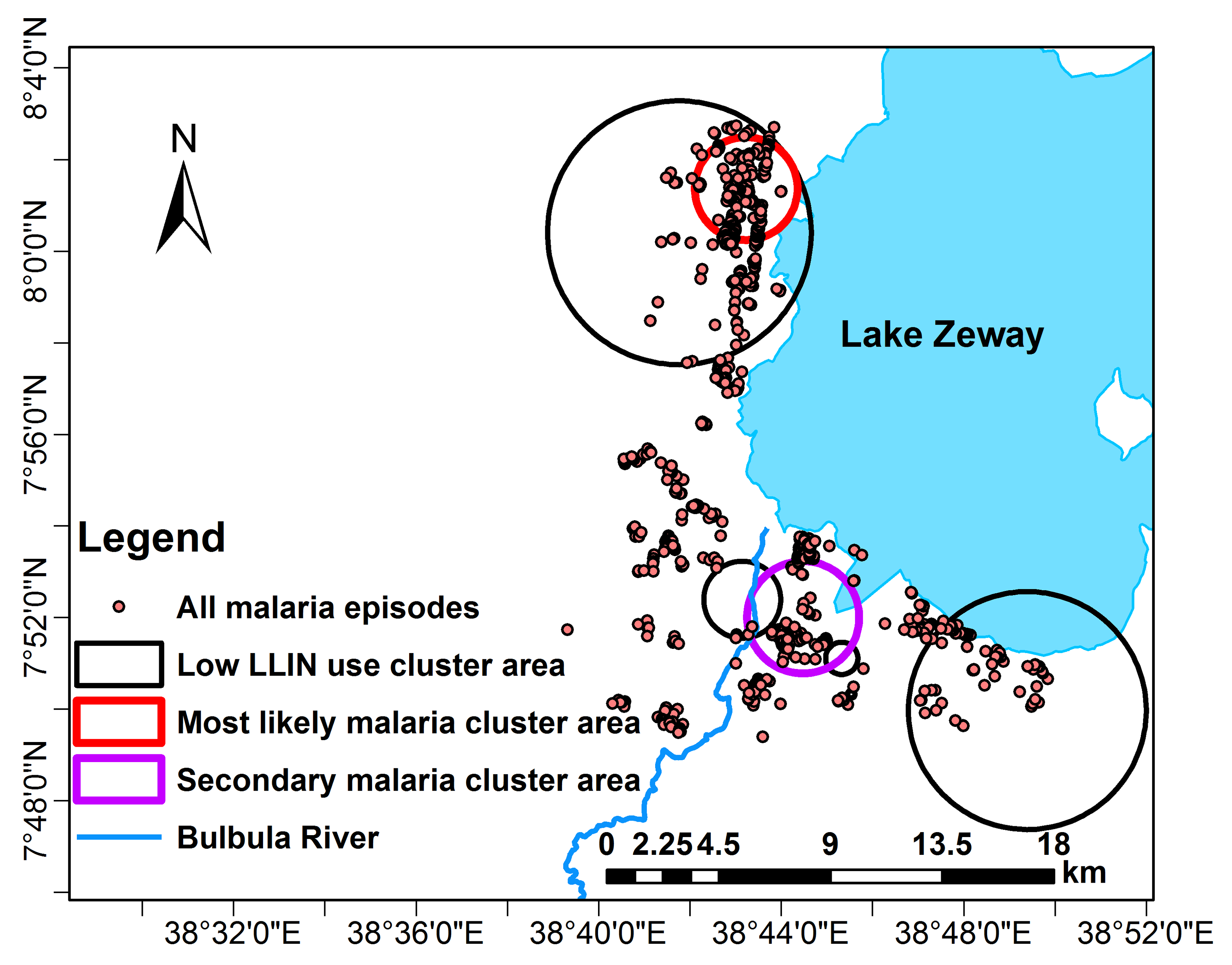

Supplement: S2 Fig — (TIF) [file pone.0222986.s007.tif]

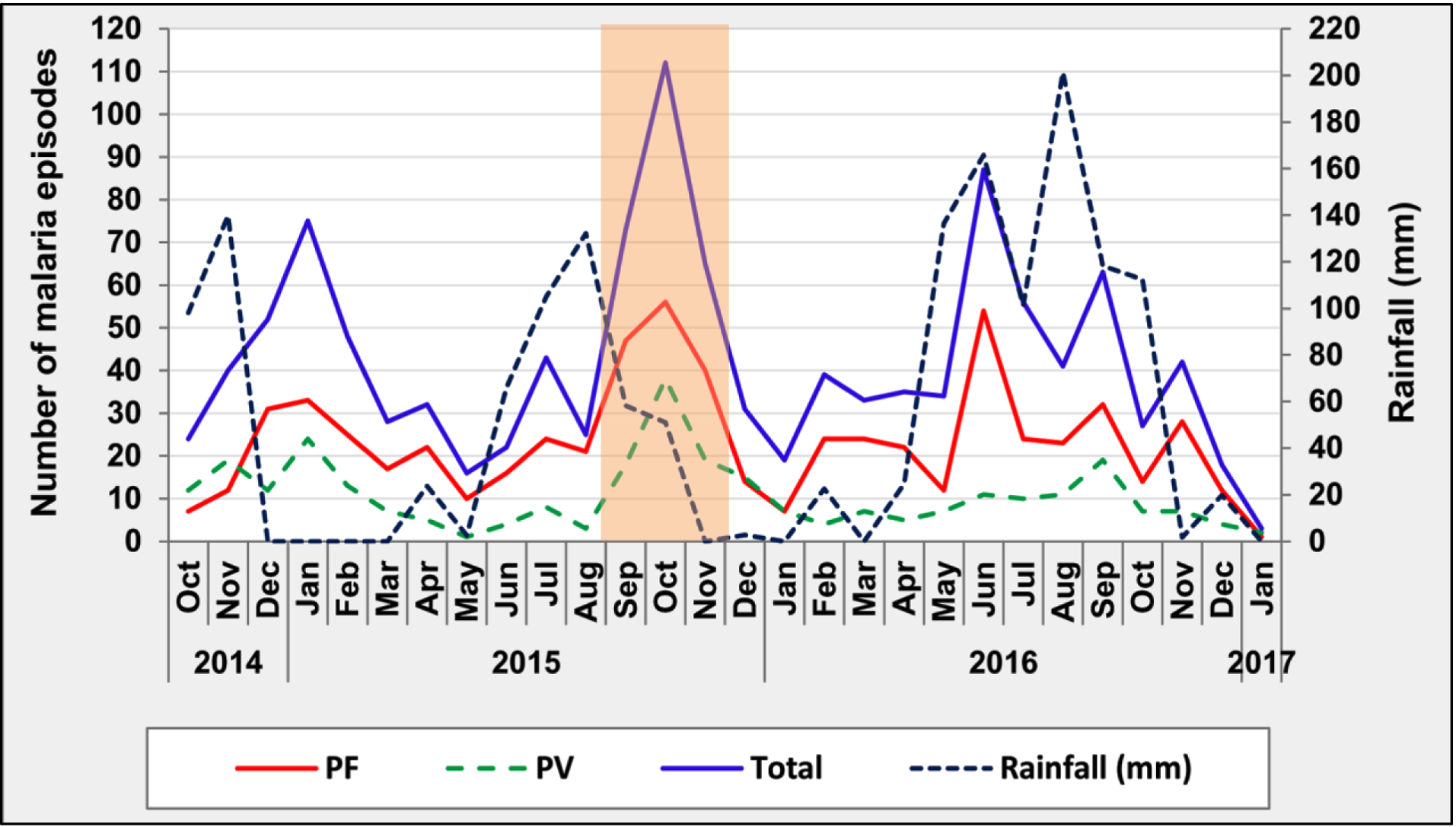

Supplement: S3 Fig — (TIF) [file pone.0222986.s008.tif]
