# Supplementary material for: Spatiotemporal clustering of malaria in southern-central Ethiopia: A community-based cohort study
Source: PLoS One. 2019 Sep 30;14(9):e0222986. doi: 10.1371/journal.pone.0222986 (PMC6768540; doi:10.1371/journal.pone.0222986)
Supplement: S1 File — (DOCX) [file pone.0222986.s009.docx]

**Construction of wealth index**

Principal component analysis (PCA) was done using 14 variables to construct wealth index. Among these variables twelve variables were binary and the other two variables were dichotomized into meaningful categories (Table 1). The Kaiser-Meyer-Olkin measure of sample adequacy was 0.79. The total variance explained by the first principal component and the corresponding Eigen value was 23.6% and 3.3, respectively. Table 2 shows frequencies, communalities, and correlations.

**Table 1. Variables and assigned values**

| **S. No** | **Variables (n=6071)** | **Assigned value** |
| --- | --- | --- |
| 1 | Electricity | Present = 1, Absent = 0 |
| 2 | Radio | Present = 1, Absent = 0 |
| 3 | Television | Present = 1, Absent = 0 |
| 4 | Mobile telephone | Present = 1, Absent = 0 |
| 5 | Chair | Present = 1, Absent = 0 |
| 6 | Table | Present = 1, Absent = 0 |
| 7 | Bed | Present = 1, Absent = 0 |
| 8 | Bicycle | Present = 1, Absent = 0 |
| 9 | Any land used for agriculture | Present = 1, Absent = 0 |
| 10 | Separate kitchen from living house | Present = 1, Absent = 0 |
| 11 | Livestock | Present = 1, Absent = 0 |
| 12 | Animal cart | Present = 1, Absent = 0 |
| 13 | Main material of the roof | Corrugated iron or cement or concrete = 1, Thatch or leaf = 0 |
| 14 | Main material of the wall | Wood with mud or wood with mud and cement = 1, No wall or only wood = 0 |

**Table 2. Frequencies of the dichotomized variables, communalities, and correlations with the first component**

| **S.No** | **Variables (n=6071)** | **Number (%)** | **Communalities** | **Correlations with the first component** |
| --- | --- | --- | --- | --- |
| 1 | Electricity | 1296 (21.4) | 0.652 | 0.436 |
| 2 | Radio | 1901 (31.3) | 0.434 | 0.477 |
| 3 | Television | 249 (4.1) | 0.703 | 0.427 |
| 4 | Mobile telephone | 3640 (60.0) | 0.442 | 0.530 |
| 5 | Chair | 4293 (70.7) | 0.495 | 0.586 |
| 6 | Table | 2108 (34.7) | 0.460 | 0.659 |
| 7 | Bed | 4257 (70.1) | 0.451 | 0.519 |
| 8 | Bicycle | 1398 (23.0) | 0.338 | 0.487 |
| 9 | Any land used for agriculture | 5606 (92.4) | 0.603 | 0.344 |
| 10 | Separate kitchen from living house | 3128 (51.5) | 0.619 | 0.630 |
| 11 | Livestock | 5000 (82.4) | 0.592 | 0.212 |
| 12 | Animal cart | 1150 (18.9) | 0.679 | 0.616 |
| 13 | Main material of the roof | 3110 (51.2) | 0.361 | 0.339 |
| 14 | Main material of the wall | 1224 (20.2) | 0.652 | 0.436 |
